# Latent Regression Analysis Considering Student, Teacher, and Parent Variables and Their Relationship with Academic Performance in Primary School Students in Chile

**DOI:** 10.3390/bs13060516

**Published:** 2023-06-19

**Authors:** Sonia Salvo-Garrido, José Zayas-Castro, Karina Polanco-Levicán, José Luis Gálvez-Nieto

**Affiliations:** 1 Departamento de Matemática y Estadística, Universidad de La Frontera, Temuco 4780000, Chile; 2 Department of Industrial and Management Systems Engineering, University of South Florida, Tampa, FL 33612, USA; josezaya@usf.edu; 3 Programa de Doctorado en Ciencias Sociales, Universidad de La Frontera, Temuco 4780000, Chile; k.polanco01@ufromail.cl; 4 Departamento de Trabajo Social, Universidad de La Frontera, Temuco 4780000, Chile; jose.galvez@ufrontera.cl

**Keywords:** latent regression analysis, self-efficacy, academic expectations, academic performance, bullying, SIMCE

## Abstract

Academic performance in primary students is fundamental to future school success; however, simultaneous analysis of different key individual, family, and teaching factors must be considered to improve understanding and benefit the development of students’ potential. This article presents a latent regression analysis model that examines the relationship between the latent variables (self-efficacy, interest in reading, bullying, parental expectations, and discrimination/exclusion, and teacher violence/aggression) and the academic performance of first-cycle primary students. The model investigates the impact of the latent variables on the standardized endogenous variables of SIMCE Mathematics and Language test scores using a quantitative, non-experimental, correlational, and cross-sectional design. The study involved 70,778 students (53.4% female), with an average age of 9.5 years (SD = 0.6), from Chilean public (33.6%) and subsidized (66.4%) schools. The results indicate that the model accounted for 49.8% and 47.7% of the mean variability in SIMCE Mathematics and Language test scores, respectively. The goodness-of-fit indices demonstrated satisfactory fits for both models. In both tests, student self-efficacy emerged as the most significant factor explaining test score variability, followed by parental expectations. Bullying was identified as a relevant factor in reducing mean performance on both tests. The findings suggest that education decision makers should address these issues to improve student outcomes.

## 1. Introduction

Several studies have demonstrated the link between students’ personal and social characteristics and academic performance, as noted in the standardized test scores in Chile and other Latin American countries [1,2,3,4,5]. This region has the highest segregation by socio-economic level [6], which affects students’ academic development. It also negatively impacts teachers and students’ families, linking with the families’ low expectations [6,7]. Specifically, Chile has a highly segregated education system [7], and with it, other conditions of psycho-social adversity are shown to be prevalent among students and have been the subject of different studies for decades, with the observation that they hinder learning and socialization, limiting students’ potential [6,8,9,10,11,12,13].

To understand the factors associated with academic performance in vulnerable populations, studies have shifted focus toward identifying individual and environmental variables related to academic success [14,15,16,17,18]. Understanding the factors that reinforce or hinder the educational development of vulnerable populations is crucial for creating equitable conditions and ensuring that all students have adequate opportunities for future success [19,20,21]. Protective and risk factors have been identified in socio-educational development processes that influence student performance and stability over time [22,23,24,25,26]. In addition, a consensus has been established regarding the reciprocal influence between individuals and the environment in these processes [16].

In this sense, self-efficacy (understood as the perception of self-efficacy) is considered an individual characteristic that reflects self-confidence in one’s cognitive capacities and internal attributes related to learning and performance [27]. According to Bandura [28], self-efficacy refers to the judgments a person makes about their own ability and their chances of achieving positive results by virtue of the proposed objectives. This influences their behavior, thoughts, and emotions, as well as their decisions regarding the effort they will invest in a particular task and persistence in the face of obstacles [28]. Self-efficacy has been studied in several fields, including mathematics, where it has been noted that self-efficacy is a significant factor in mathematics performance in arithmetic and problem-solving tasks for both students and teachers, being demonstrated in cross-sectional and longitudinal studies [29,30,31,32,33,34].

Studies have also identified other factors that contribute to satisfactory math performance, including gender, age, school resources, and socioeconomic level, as well as the student’s motivation for a certain activity [33,35,36,37,38]. Fisher et al. [39] studied the relevance of gender in math performance and found that the low number of women in science, technology, engineering, and mathematics (STEM) disciplines is mainly due to their lower degree of self-efficacy. Consequently, it is important to consider the gender construct when studying performance in mathematics, as it is a culturally stereotypical domain [40,41,42,43]. For example, Borgonovi and Pokropek [44] found higher means for boys than girls in the perception of self-efficacy in mathematics, suggesting the need to consider and control for the influence of the gender variable in the link between academic performance and self-efficacy [29].

Self-efficacy in language is understood as the students’ perceptions of their abilities and possibilities of achieving a suitable performance in this specific area [45], while interest in reading is defined as the level of pleasure or enjoyment taken from reading a text [46,47]. When established in early development, the perception of self-efficacy in language and interest in reading are considered important factors for coping with the difficulties that can arise at school [24,48,49]. In addition, the ability to read is also associated with attitudes toward autonomy, i.e., with undertaking activities in this area alone and asking for help only when required, as well as toward the use of information and communication technologies to reinforce these skills [50,51,52,53].

Bullying is defined as a behavior of intentional and repeated aggression toward a victim over time [54]. Traditional bullying and cyberbullying are linked to academic performance, acting as precursor and consequence [55,56]. Moreover, bullying can affect the adjustment capacities and social efficacy of victims [17,57,58,59]. In the case of cyberbullying, a sense of self-efficacy is crucial to developing defensive behaviors against cyber-attacks [60,61]. Finally, it is worth noting that teachers sometimes observe bullying and aggression between students, which negatively affect the teaching–learning process, social relations generated in the class, and academic performance [62].

On the other hand, the link between family and school is also relevant to expectation development [63,64], with a sense of commitment to family obligations increasing students’ motivation to study [65,66] and parental and community emotional support reinforcing the academic capacities of children with difficulties in the socio-affective domain [67,68,69]. In addition, parental involvement, particularly from the mother, and parental expectations can reduce the negative and stressful effects of adverse socio-affective and contextual conditions [70,71], which are predictors of school performance, along with a positive school climate and resilient teachers [72,73].

Gutiérrez et al. [74] found that permanent family and teacher support contribute to school involvement and the satisfaction of youths. Ansong et al. [75] emphasized the importance of encouraging students regarding academic expectations and a prospective view of life for satisfactory school performance. Randall and Burley [76] suggest that teachers’ self-efficacy beliefs influence their commitment to their profession, impacting their contribution to students’ academic processes. Bonneville-Roussy et al. [77] suggest that cultural differences affect the extent of teachers’ self-efficacy development on youths’ self-efficacy in teaching practices. Miller et al. [78] indicate that teachers’ sense of self-efficacy is associated with the competence perceived by the students and the respect perceived by the teachers. Theron and Theron [79] argue that good academic performance requires an active connection between the teacher, family, community, and the student’s ability to respond.

The preceding provides evidence of the relevance of considering factors from various domains (personal, teachers, and family) to analyze the relationship with primary students’ academic performance. This is based on previously conducted studies [24,80] that demonstrate, for example, the importance of the variable self-efficacy and its connection to academic performance. In addition, it is considered that standardized mathematics and language tests assess knowledge and skills required in other areas of learning [24,80]. Hence, this study uses a latent regression model to explore the association between variables related to the student (self-efficacy in mathematics, self-efficacy in language, and interest in reading), teachers (Violence/Aggression), and parents (Parents’ expectations, Discrimination/Exclusion) to academic performance on the SIMCE tests in mathematics and language of students in the first cycle of public and private-subsidized primary schools in contexts of significant family and social vulnerability in Chile. Therefore, the following research question is posited: What are the relations between the latent variables of students, teachers, families, and the academic performance of first-cycle primary students in Chile?

## 2. Materials and Methods

### 2.1. Participants

A total of 70,708 fourth-grade primary students (46.6% male and 53.4% female) in Chile who took the SIMCE tests in 2015 were considered in this study, representing 46% of the population. As a criterion of student inclusion, it was believed that there were no missing values in the analysis variables. Regarding place of residence, 90.3% of the students reported living in urban settings (9.7% rural). Considering the type of school, 23,780 (33.6%) children belonged to public schools, and the remaining (66.4%) to private-subsidized schools. The mean age of the sample was 9.5 (SD = 0.6), with a minimum of 9 and a maximum of 10 years old.

### 2.2. Instruments

The census SIMCE tests evaluate the learning of all students in Chile, in relation to the current national curriculum, in different subjects and school levels, to contribute to improving the quality and equity of education. In addition, it collects information from students, teachers, and parents/guardians, through context questionnaires about Chilean education [81].

### 2.3. Data

The database of the SIMCE mathematics and language test results of fourth-grade primary students from 2015 and the context questionnaires provided by the Chilean Education Quality Assurance Agency were used under strict confidentiality. For more information, see https://www.agenciaeducacion.cl/ (accessed on 10 March 2022).

### 2.4. Procedure

The processes for applying the instruments (tests and context questionnaires) were carried out according to instructions, manuals, and action protocols based on the international standardization criteria defined in the document Standards for Educational and Psychological Testing [82]. Such documents are intended to provide equivalent conditions for all students when taking the test, while ensuring confidentiality of the information and safeguarding the materials applied [81].

With respect to the application of contextual tests and questionnaires, the examiner reviews the material received by the Education Quality Assurance Agency and reports possible inconsistencies. Next, the classroom is prepared based on the seating arrangement and booklet allocation criteria established in the Application Manual. Then, the students are instructed to enter the classroom, attendance is taken, and the general instructions for each test, contained on the cover of booklets, are read aloud. These instructions are key to the students being familiar with the general conditions before taking the test. Once the test is finished, the material is carefully collected, packaged, and the box containing the test is sealed for dispatch to the corresponding collection center [81].

### 2.5. Method

This is a quantitative, non-experimental, correlational, and cross-sectional study [83]. A latent regression analysis [84] was carried out considering covariates, where the endogenous variables were the SIMCE Mathematics and Language test scores, with seven variables exogenous. Six latent variables comprised 24 items measured with an ordinal scale of four categories (1 = Never/Very untrue, 2 = Few times/Untrue, 3 = Many times/True, 4 = Always/Very true). They were distributed as follows: L1: Interest in reading, made up of four items; L2: Bullying, made up of eight items; L3: Self-efficacy in mathematics, made up of three items; L4: Self-efficacy in reading, made up of three items; L5: Violence perceived by parents, made up of four items; L6: School discrimination perceived by teachers, made up of four items; and standardized parental educational expectations, corresponding to the perception of the highest level of education that the student would be able to complete in the future, and the covariates included were gender and type of school. SEMs (Structural Equation Model) with latent variables allow us to specify regression analyses at the level of latent variables (factors) that are corrected for measurement error. This procedure has the advantage that error of measurement can be taken into account explicitly for both the independent and the dependent variable(s). Furthermore, SEMs with latent variables allow us to estimate the reliabilities of the manifest variables. The explicit consideration of measurement error leads to a more precise estimation of the parameters of the regression model compared to manifest regression analyses with observed variables that are not adjusted for measurement error. Multiple indicators are required for each latent variable in a latent regression model (LRM) [84].

To estimate the models, the polychoric correlation matrix [85] was used along with the method of weighted least squares means and variance adjusted (WLSMV) [86]. The following goodness-of-fit indicators were used to assess the quality of the models: comparative fit index (CFI), Tucker–Lewis index (TLI), root mean square error of approximation (RMSEA), and standardized root mean square residual (SRMR). An SRMR value of <0.08 is considered a good fit [87]; a CFI and TLI value of ≥0.90 is regarded as a reasonable fit and ≥0.95, an excellent fit [88]; and an RMSEA value of < 0.08 is considered a reasonable fit [87], and ≤0.06, an excellent fit [89]. The reliability of each latent variable was determined using the McDonald’s Omega coefficient [90], which must be between 0.70 and 0.90 to be acceptable [91].

**Figure 1:**
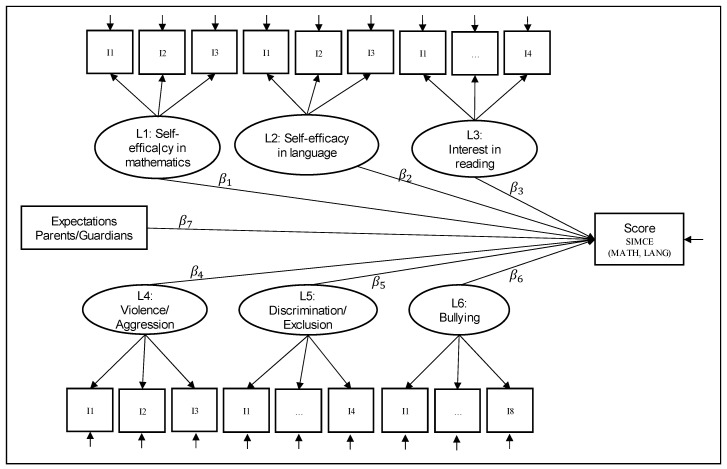
Latent regression analysis on the SIMCE mathematics and language scores. Where: I1,…,I8 represent the items of each of the latent factors and βi,i = 1,…,6 are the parameters of the model to be estimated.

Figure 1 shows the LRM to be estimated. All analyses were performed with the Mplus program, version 8.3 [92].

## 3. Results

Table 1 presents the descriptive statistics, mean and standard deviation, of the standardized variable scores by school type and gender. The means of the standardized scores of the endogenous variables are below the mean of the overall standardized score in public schools, regardless of gender. However, in private-subsidized schools, these values are above the mean. For example, boys have a higher mean in math self-efficacy (L1) than girls. On the other hand, in language self-efficacy (L2), only girls from private-subsidized schools are above the overall mean; boys from school public have the highest mean in bullying. On the other hand, parents/guardians at private-subsidized schools have above-mean academic expectations for their children.

Two LRMs were adjusted for the endogenous variables SIMCE mathematics (M1) and language (M2) scores. For M1, the goodness-of-fit indices had the following values: WLSMV-χ2 (304) = 70,933.2, *p* < 0.001; CFI = 0.937; TLI = 0.928; RMSEA = 0.057 (90%C.I. = 0.057, 0.058); SRMR = 0.068. These values indicate that the proposed M1 model fits the data reasonably. R^2^ = 0.498, 49.8% of the variability of the SIMCE mathematics score is explained by the exogenous variables. For M2, the goodness-of-fit indices had the following values: WLSMV-χ2 (304) = 71,542.4, *p* < 0.001; CFI = 0.937; TLI = 0.927; RMSEA = 0.058 (90%C.I. = 0.057, 0.058); SRMR = 0.069. These values indicate that the proposed M2 model fits the data reasonably. R^2^ = 0.477, 47.7% of the variability of the SIMCE language score is explained by the exogenous variables. Both R^2^ values are greater than 0.3. In social and behavioral science applications, the R^2^ value is usually not higher than 0.3 because relevant exogenous variables that are difficult to measure may not be included in the model [93]. These results show the relevance of considering, on the one hand, a latent regression analysis, and on the other, considering the variables gender and type of school as covariates.

Table 2 shows the standardized factor loadings, all greater than 0.64. They are therefore considered adequate to define the latent factor. For the latent variable self-efficacy in mathematics, the omega coefficient was 0.85; self-efficacy in language was 0.74; interest in reading was 0.84; violence/aggression was 0.87; discrimination/exclusion was 0.89; and bullying was 0.92. These reliability values are considered acceptable for all factors.

Table 3 shows the values of the standardized estimated coefficients of the LRM for M1 and M2. All the estimated coefficients were statistically significant, *p* < 0.001. For M1, the most relevant variables that explain a positive change in the SIMCE score were self-efficacy in mathematics, self-efficacy in language, expectations (parents/guardians), and bullying for a negative change. For M2, they were self-efficacy in language, expectations (parents/guardians), and bullying, respectively.

## 4. Discussion

The results reveal latent factors that tend to configure a cross section of variables significantly influencing academic performance. To achieve this, a latent regression analysis was adjusted to the measurement scale of the items and their interrelationships with excellent indicators of fit. For M1, self-efficacy in mathematics was the most relevant latent factor to explain the mean variability of the SIMCE mathematics test scores, followed by self-efficacy in language and parent/guardian expectations. By contrast, bullying and parent/guardian discrimination/exclusion were the most relevant factors to decrease, in the mean, this performance. For M2, self-efficacy in Language was the most relevant latent factor to explain the mean variability of the SIMCE language test scores, followed by parents’/guardians’ expectations and self-efficacy in mathematics. On the contrary, bullying and parent/guardian discrimination/exclusion were the most relevant to decrease, in the mean, this performance. These results present an important degree of theoretical consistency with previous studies in terms of the prevalence of the self-efficacy factor in mathematics [25,29,30,31,33,34,37,57], and indicate that this internal attribute is associated with the learning processes responsible for satisfactory academic performance. In the field of language, the self-efficacy factor proves to be a relevant variable related to the development of language skills and interest in reading [24,53,94]. In this sense, the predominance of the level of self-efficacy as a protective factor of the student body is consistent with Salvo-Garrido et al. [27] and Anghel [26], who emphasize the need to develop or reinforce students’ confidence in their own cognitive skills, which will allow them to overcome the obstacles that arise at school and in their life in general. Similarly, the relationship established by Pitzer and Skinner [95] between self-efficacy and self-appreciation as factors that influence mathematics performance is interesting.

In the same vein, the literature tends to conceive the perception of self-efficacy as an individual internal characteristic, so it could be argued that confidence in one’s abilities is significantly associated with socio-educational and affective relationships, which the student has or does not have the chance to develop [96]. Semanchin et al. [96] and Salvo-Garrido et al. [26] refer to the significant emotional support offered to the student by adults or professional figures as benefitting their education outcomes. This would increase the capacity for self-regulation and self-directed learning, factors that have been linked to the variable of self-efficacy [97,98]. In this sense, the emergence of expectations also theoretically refers to a positive socio-affective context anchored in the family [71] and teaching dimensions [32]. According to several studies, this factor (expectations) contributes to youths’ increased motivation for study, greater involvement and school satisfaction, and the development of a prospective view of life. These are all socio-affective processes that can have a significant impact on academic performance [17,24,26,68,74,75].

However, latent factors that allude to bullying, discrimination, exclusion, and the deployment of aggressive behaviors, whether they are perpetrated in person or virtually [60], also constitute risk variables that need to be addressed in the development of educational strategies, reinforcing the sense of self-efficacy [61]. Specifically, bullying is recognized as a major problem that affects children’s academic performance all over the world, impacting their academic commitment and class attendance [56]. Moreover, while students who are spectators of cyberbullying and traditional bullying are key to reducing the aggressions and consequences for the victim, their actions will depend on how confident they feel about their ability to stop the aggressions on a social and emotional level [61]. Generally, aggressions experienced and observed as well as the difficulties schools have in managing good interpersonal relations affect the coexistence of the members of the education community and, therefore, academic performance [55]. The relevance that students involved in bullying (online and offline) receive social support from teachers and other students, offering them prompt attention, is highlighted, adding that preventive actions are carried out constantly, promoting suitable behavioral adjustment and appropriate interpersonal relationships [55]. In addition, regulations in the schools need to be effective in protecting children from aggression phenomena, while at the same time applying laws and policies [56] that favor the optimal development of the students in a safe environment [17,26,57,58,59].

Concerning the findings linked to the perception of self-efficacy and gender differences, it may be stated that the highest mean in self-efficacy in mathematics observed in boys compared to girls could condition students’ future academic choices, clarifying that there are a small number of women in the areas of science, technology, engineering, and mathematics [39]. This could be related to gender stereotypes associated with the mathematics domain, which would culturally be associated with the skills of boys and, to a lesser degree, girls [41]. In this sense, cultural gender stereotypes are relevant to consider, since they may be at the basis of the higher means obtained by boys compared to girls in terms of their mathematics self-concept [44]. Taking into account the socioeconomic segregation that Chile and Latin America generally experience [6,7], which has a detrimental impact on academic performance on standardized tests [1,2,3,4,5], the situation becomes more complex, preventing boys, and especially girls, from reaching their full potential and lowering family expectations [6,8,9,10,11,12,13]. Consequently, it is important to make families and teachers aware of the issue so they can offer support and positive reinforcement to change this situation. In addition, it is suggested that this variable should be controlled in studies on academic performance and the development of school self-efficacy [29].

The latent factors indicated concur in academic processes as conceptual devices, but also as strategic, when developing programs that support the vulnerable student population. Thus, emphasis is placed on the need for socio-educational and affective convergence between the student, parental and educational resources, and institutional devices. Moreover, it is crucial to create a school and family climate free from violence and disqualification, with a consistent normative dimension, which can strengthen the relationship between self-efficacy and expectations while reinforcing academic provisions and the positive development of a prospective view of life in each boy and girl who must continually face the vicissitudes of adversity. The evidence suggests that education decision makers should include efforts to address this issue in the education community.

Finally, future lines of research are proposed to explore the longitudinal relationships among the constructs addressed in this study. In addition, it would be relevant to include the analysis of school climate in light of its association with several variables, such as cyberbullying and academic performance, among others. As for the limitations of this study, it is important to emphasize that the results obtained are cross-sectional, which provides limited evidence in terms of temporality and causality. Moreover, it is worth noting that the context questionnaires comprising the SIMCE are based on self-reported responses, which implies that the results reflect the participants’ perceptions and have not been contrasted with other data sources.

## Figures and Tables

**Table 1:** Descriptive statistics, mean, and standard deviation.

School	Gender	Lang	Math	L1	L2	L3	L4	L5	L6	Expect.
Public	Male	−0.36 (1.03)	−0.30 (1.04)	0.02 (0.66)	−0.09 (0.49)	−0.05 (0.61)	0.23 (0.80)	0.18 (0.57)	0.26 (0.70)	−0.36 (1.18)
	Female	−0.18 (0.99)	−0.35 (1.01)	−0.07 (0.64)	−0.04 (0.49)	0.15 (0.59)	0.20 (0.80)	0.17 (0.60)	0.07 (0.70)	−0.30 (1.15)
Private-subsidized	Male	0.06 (1.00)	0.20 (0.95)	0.04 (0.66)	−0.01 (0.49)	−0.16 (0.62)	−0.04 (0.70)	0.07 (0.52)	0.07 (0.63)	0.15 (0.88)
	Female	0.20 (0.94)	0.13 (0.93)	−0.07 (0.67)	0.04 (0.48)	0.05 (0.62)	−0.08 (0.70)	0.04 (0.53)	−0.12 (0.61)	0.18 (0.84)

Expect. = Expectations parents/guardians, (SD). Note: The mean differences by school type and gender were statistically significant, *p* < 0.001.

**Table 2:** Description of the latent factors and their respective standardized factor loadings.

Latent Variable	Items	Est.	SE	*p*-Value
Self-efficacy in mathematics	Doing math problems is entertaining.	0.742	0.002	<0.001
	I do better in math than my classmates.	0.749	0.002	<0.001
	I learn math easily and quickly.	0.919	0.002	<0.001
Self-efficacy in language	Reading is easier for me than for many of my classmates.	0.656	0.003	<0.001
	Reading is easier for me than any other class activity.	0.736	0.003	<0.001
	I am simply good at reading.	0.687	0.003	<0.001
Interest in reading	I would be happy if someone gifted me a book.	0.726	0.002	<0.001
	I would like to have more time for reading.	0.774	0.002	<0.001
	I like reading.	0.869	0.002	<0.001
	I read for fun.	0.649	0.003	<0.001
Violence/Aggression	Insults, slurs, derision, and disqualifications among students.	0.899	0.002	<0.001
	Threats or harassment among students.	0.878	0.002	<0.001
	Students break or damage the establishment.	0.710	0.003	<0.001
Discrimination/Exclusion	Gender of the student.	0.760	0.004	<0.001
	Physical characteristics of the student.	0.869	0.002	<0.001
	Personality of the student.	0.741	0.003	<0.001
	Way of dressing or physical appearance of the student.	0.881	0.003	<0.001
Bullying	Some school students have stolen from another classmate.	0.759	0.002	<0.001
	Some school students have scared another classmate and forced them to do things they did not want to.	0.842	0.002	<0.001
	Some school students have threatened to beat another classmate.	0.827	0.002	<0.001
	Some school students have forced another classmate to give them their belongings or their money.	0.862	0.002	<0.001
	Some school students have intentionally broken another classmate’s belongings.	0.807	0.002	<0.001
	Some school students have sent messages to annoy or threaten another classmate.	0.740	0.003	<0.001

Est. = Estimate, SE = Standard error.

**Table 3:** Standardized coefficient estimates of the latent regression model.

		Mathematics			Language		
Variable	Participant	Est. Coef.	SE	*p*-Value	Est. Coef.	SE	*p*-Value
Intercept		0.039	0.005	<0.001	−0.253	0.007	<0.001
Self-efficacy in mathematics	Student	0.397	0.004	<0.001	0.203	0.004	<0.001
Self-efficacy in language	Student	0.309	0.004	<0.001	0.435	0.004	<0.001
Interest in reading	Student	0.034	0.004	0.031	0.099	0.004	<0.001
Expectations	Parents/guardians	0.280	0.003	<0.001	0.266	0.004	<0.001
Violence/Aggression	Teacher	−0.158	0.004	<0.001	−0.144	0.004	<0.001
Discrimination/Exclusion	Parents/guardians	−0.182	0.005	<0.001	−0.168	0.004	<0.001
Bullying	Student	−0.251	0.004	<0.001	−0.297	0.004	<0.001
Gender (Female = 1, Male = 0)		−0.056	0.004	<0.001	0.014	0.004	0.001
Type of school (Private-subsidized = 1, Public = 0)		0.048	0.004	<0.001	−0.001	0.004	0.735

Est. coef = Estimate coefficient; SE = Standard error.

## Data Availability

The data presented in this study are available on request from the Ministry of Education of Chile.
